# Exploring Cancer Patients’ and Caregivers’ Perspectives and Knowledge Regarding Biomarker Testing in Canada

**DOI:** 10.3390/curroncol32060292

**Published:** 2025-05-22

**Authors:** Patil Mksyartinian, Selina Xu, Chrissa Barroma, Sandra Peláez, Barry D. Stein

**Affiliations:** 1 Colorectal Cancer Canada, Westmount, QC H3Z 2P9, Canada; patilm@colorectalcancercanada.com; 2 School of Public Health Sciences, University of Waterloo, Waterloo, ON N2L 3G1, Canada; sy5xu@uwaterloo.ca; 3 School of Population and Global Health, McGill University, Montreal, QC H3A 0G4, Canada; chrissa.barroma@mail.mcgill.ca; 4 Ingram School of Nursing (ISoN), McGill University, Montreal, QC H3A 0G4, Canada; sandra.pelaez@mail.mcgill.ca; 5 Research Centre of Sainte-Justine University Hospital (RC-CHUSJ), Montreal, QC H3T 1C5, Canada

**Keywords:** biomarker testing, precision medicine, colorectal cancer, genomic sequencing, cancer, cancer outcomes, tumour, patient-centred care

## Abstract

While biomarker testing can provide various benefits for cancer patient outcomes, numerous challenges persist that cause inequities in access across Canada. An online survey consisting of 51 questions was disseminated to evaluate biomarker testing and precision medicine knowledge and experiences from Canadian patients and caregivers. Responses were recorded between June 2023 and January 2024 and assessed various aspects of the biomarker testing experience including the expectations and challenges of patients. Quantitative and qualitative analyses were conducted using Microsoft Excel and R for descriptive and correlative data analysis, respectively. Among the 74 responses, patients reported an overall moderate experience with positive outcomes for those who underwent biomarker testing, including changes to treatment plans and the shrinking of tumours. The main challenges identified included knowledge gaps, a lack of testing availability, turnaround time for results, and financial constraints, all of which contribute to the disparities in biomarker testing access. Qualitative analysis of responses further emphasized a strong patient desire for patient-centred care and collaborative decision-making for biomarker testing options and treatment planning. Addressing these challenges through increased education, policy advocacy, and advancing infrastructure can help to reduce interprovincial inequities in biomarker testing and contribute to improving cancer patient outcomes.

## 1. Introduction

In recent years, there have been consistent population-level improvements in Canadian cancer care outcomes, with considerable decreases in mortality and incidence accompanied by increased cancer survival. However, cancer continues to be the leading cause of death in Canada, with one in four Canadians estimated to have died from this disease in 2024 [1]. Cancer biomarkers have transformed cancer care, enabling personalized treatment based on a tumour’s molecular profile, which has led to improvements in cancer therapeutics and patient prognosis [2]. Biomarkers are biological patterns found in blood, other bodily fluids, or tissues that can indicate normal or abnormal processes and/or the presence or absence of a disease or condition [3]. They can also be used to examine how effectively the body responds to a treatment for a disease or condition [3]. Biomarker testing (also known as genomic profiling or tumour genetic testing) examines genes, proteins, and other substances and provides important information about an individual’s unique cancer profile [4]. Biomarker testing allows for the determination of the most appropriate treatment based on an individual’s genetic profile, which maximizes treatment effectiveness and minimizes side effects [4]. Biomarker testing can also be used to monitor treatment response, detect disease progression, and help avoid unnecessary treatment, leading to potential cost savings for patients and the healthcare system [4].

Biomarker testing plays an important role in the growing field of personalized medicine (also known as precision medicine), which is an approach to cancer care in which the diagnosis and treatment are tailored based on an individual’s genetic profile and other biological markers [4,5]. Personalized medicine is a promising avenue through which biomarker tests are used to guide disease characterization and provide early and accurate cancer diagnoses [5].

Biomarker testing applications in cancer care can be prognostic, diagnostic, predictive, and pharmacodynamic [2]. Prognostic biomarkers can inform the likelihood of a cancer outcome independent of any treatment received [6]. For instance, breast cancers with human epidermal growth factor receptor 2 (HER2) expression have a poorer prognosis, while for colorectal cancer (CRC), tumours with high levels of microsatellite instability (MSI) have a better prognosis [7]. Diagnostic biomarkers can detect or confirm the presence of a disease or condition and can provide further information as to the subtype of the disease [8]. For example, the carcinoembryonic antigen (CEA) biomarker is commonly utilized in the diagnosis and monitoring of CRC [7]. Predictive biomarkers play an important role in companion diagnostics and can predict how a cancer will respond to a certain treatment [2]. In CRC, microsatellite instability-high (MSI-H) or deficient mismatch repair (dMMR) status is a predictive biomarker and can determine response to immune checkpoint inhibitors, such as pembrolizumab and nivolumab [7]. Pharmacodynamic biomarkers can help anticipate the response to a treatment, such as chemotherapy. 5-Flurouracil (5-FU), a staple in colorectal cancer chemotherapy regimens, is associated with negative side effects (i.e., diarrhea, cardiac toxicity, myelosuppression, etc.) in nearly 30% of recipients [9]. A deficiency in Dihydropyridimine dehydrogenase (DPYD), a type of biomarker, was found to be associated with 5-FU toxicity [10].

As demonstrated, biomarker testing is a powerful tool for cancer detection, prognostic insight, and individualizing and monitoring cancer treatment. Biomarker testing has played an important role in improving overall cancer survival, increasing disease-free progression, enhancing quality of life, and reducing the risk of cancer recurrence [11,12]. Further, biomarker testing may reveal patient eligibility for participation in clinical trials investigating novel therapies—increasing patients’ treatment options [12].

Although, despite the various advantages of accessing biomarker testing for cancer care, there are various systematic barriers in Canada, such as the Canadian biomarker testing infrastructure, awareness of biomarker testing, and access to biomarker testing. Canadians are hindered from accessing biomarker testing due to systemic roadblocks such as a lack of financial support from provincial health ministries and a lack of resources in the forms of administration, testing infrastructure, and human resources [11,13,14]. For example, in Ontario, the Comprehensive Cancer Biomarker Testing Program offers biomarker testing, but the testing is limited to specific testing sites and is only available for certain cancer types and testing indications (e.g., KRAS testing for patients with advanced CRC) [15]. Consequently, the guidelines for delivering and reimbursing biomarker tests vary across the provinces and territories in Canada, as each province has different processes for considering what type of biomarker tests to reimburse and different performance and quality standards [16]. These challenges are also exacerbated by testing capacity barriers, as not all provinces have the same capacity to deliver biomarker testing or reimburse the same type of tests [16]. Canadian laboratories are frequently underequipped to support biomarker testing or cannot afford to perform regular diagnostic tests [17]. Larger provinces such as Ontario, Quebec, Alberta, and British Columbia have more than one testing facility to deliver genomic testing, but they lack regional analytic standards which leads to test performance variations across provinces [16].

Moreover, drug costs are continuing to increase as a larger number of cancer patients receive targeted therapies and immunotherapies, which rely on biomarker testing for access [18]. This can be challenging for patients who have to pay out of pocket for genomic tests that are not publicly funded, which can lead to disparities in accessing future innovation through clinical trials or compassionate access programmes [17,18].

Furthermore, improving biomarker testing access requires a two-pronged awareness promotion approach—educating both patients and healthcare providers. Significant knowledge gaps (e.g., unfamiliarity with test ordering procedures) among lung cancer specialists have been associated with lower biomarker testing rates among lung cancer patients in Ontario [19]. Similarly, patients also demonstrate knowledge and awareness gaps, as some do not understand the importance and application of biomarker testing, while others are not aware of their testing options and patient support programmes [6,16].

To improve access to biomarker testing and precision medicine, it is important to understand the perspectives not only of healthcare professionals but patients and their caregivers as well. The current literature primarily reflects the perspectives of healthcare professionals, this study uniquely surveys patient and caregiver views and provides a distinctly Canadian patient perspective on the experiences and challenges associated with biomarker testing [20,21,22,23]. The aim of this study is to explore cancer patients’ and caregivers’ knowledge, awareness, and expectations of biomarker testing, as well as to identify the benefits and difficulties they encountered during the process. Our findings may be conducive to promoting access to biomarker testing and personalized medicine for Canadian cancer patients, ultimately improving cancer care outcomes.

## 2. Materials and Methods

### 2.1. Target Population 

The target population of this study included cancer patients 18 years or older, who were either undergoing (or had undergone) treatment and their caregivers who were supporting (or supported) them during the treatment process.

### 2.2. Survey Design and Development 

A cross-sectional study to characterize Canadian patients’ and caregivers’ experiences with biomarker testing was conducted. A 51-item, multiple-choice, and free-text responses online survey was created using SurveyMonkey. The survey consisted of three sections: (a) personal demographics and cancer diagnoses (where applicable), (a) biomarker testing knowledge, and (c) biomarker testing experience. To ensure the medical accuracy and relevance of the survey within the Canadian context, the questions were developed by the Colorectal Cancer Canada (CCC) team and reviewed by the ad hoc scientific advisory committee made up of oncologists and pathologists. The survey was available in both English and French.

### 2.3. Survey Administration 

The online survey was disseminated and available for completion from June 2023 to January 2024. Information on how to participate in the survey was (a) sent to cancer advocacy groups and clinical partners who invited potential participants through their email lists, and (b) shared on social media platforms such as Facebook, Instagram, and Twitter. Recruitment materials included newsletters and social media content. Informed consent was collected from each participant prior to the completion of the survey. As an incentive, survey participants who fully completed were offered entry into a random selection draw to win one of ten $25 (CAD) Amazon e-gift cards. In order to uphold participant anonymity, email addresses were collected for the purpose of entering the draw and were stored separately and destroyed upon the selectees’ remuneration.

### 2.4. Data Storage

Survey data were collected and stored on the SurveyMonkey secured server, which is compliant with Health Canada data security standards. To ensure the confidentiality of the participants, data extracted for analysis were stored on Colorectal Cancer Canada’s password-protected servers, to which access was restricted to authorized personnel. These measures were taken as outlined upon collecting informed consent.

### 2.5. Data Analysis

Data from the survey were extracted from the SurveyMonkey platform in Excel format (csv file). Quantitative data were analyzed using Microsoft Excel to analyze descriptive and inferential statistics. Descriptive statistics included the calculation of the measures of central tendency (e.g., mean, median, and mode) for continuous variables and response pool composition (percentages) for nominal variables. Microsoft Excel was also used to create visual representations of the results, using frequency tables, figures, and charts. Stratification was used to compare differences between groups (e.g., age groups, gender, and educational attainment) and identify relevant trends between specific subgroups (e.g., to identify trends regarding the familiarity of terms by age group and educational attainment).

Furthermore, R version 4.3.1 was used to conduct correlative analyses, such as Kendall’s rank correlation test, Fisher’s exact test, and Spearman’s rank correlation to assess the relevant association between categorical variables (both nominal and ordinal) in the study. Statistical significance was set a priori at *p* < 0.05. Statistical analyses were included to enhance the interpretation of the descriptive results and were determined based on the type of data available (e.g., nominal, ordinal, etc.).

Regarding qualitative analysis, French open-ended responses were translated into English, and the back-translations were approved by the CCC team prior to analysis. Qualitative analysis entailed transferring the free-text responses from the three open-ended questions and responses were correspondingly designed and analyzed with the concept of health self-management in mind [24]. Anticipating the potential nature of the responses, we (a) followed Van de Verle et al.’s (2019) conceptualization of self-management, defined as the ability to adapt and address social, physical, and emotional challenges; and (b) analyzed only two attributes—namely, the person-oriented and person-environment-oriented attributes [25]. The analysis consisted of using MAXQDA (Version 24) to thematically organize the extracted responses using the selected theoretical framework as a reference [26]. Information regarding the online survey can be found in Supplementary Materials.

### 2.6. Ethics Approval

The study received ethics approval from the Genetic Alliance Institutional Review Board (REB# MCRC001).

## 3. Results

### 3.1. Quantitative Analysis

#### 3.1.1. Demographics

Responses from participants who left more than 49 responses unanswered were removed from the final data file. Of the 87 responses collected, 13 were excluded from the final data analysis due to incomplete responses. A total of 74 survey responses were analyzed from all provinces of Canada except Prince Edward Island. Those residing in Ontario composed 40.5% of all respondents. Meanwhile, 81.1% of respondents identified as female and 18.9% identified as male. Most respondents were between the ages of 50 and 69 years (54%). Sample demographic details, including residence, gender, age ranges, educational attainment, and employment are shown in Table A1.

#### 3.1.2. Cancer Connection and Status 

Of the 74 respondents, 90.5% respondents were cancer patients and 9.5% were caregivers. A total of 37% of the responses were from patients currently undergoing cancer treatment, 34% were from patients who had previously undergone cancer treatment, and 20.3% were from people who reportedly had no evidence of the disease as determined by examination or imaging tests.

**Figure A1:**
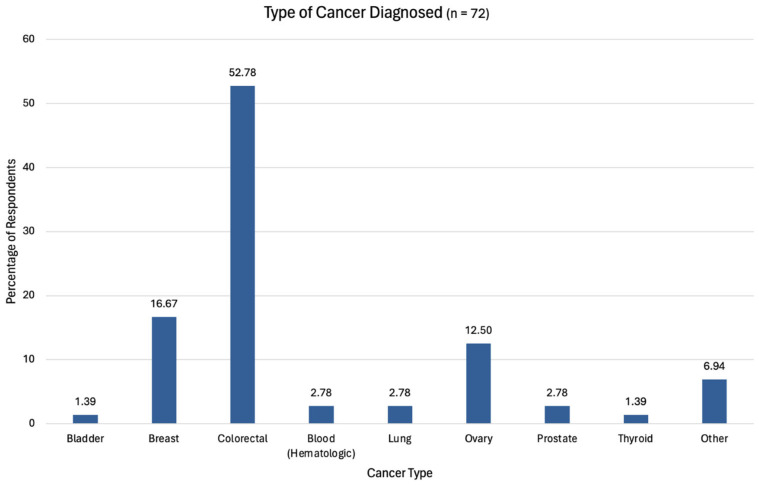
Type of cancer diagnoses. Note, “Other” cancers that were diagnosed but were not included as answer options for the multiple-choice question included myeloma, metastatic neuroendocrine, lymphatic, appendix, anus, and neuroendocrine tumours with the lung as the primary site.

Most respondents were diagnosed with colorectal cancer (CRC) (52.7%). None reported a diagnosis of brain/CNS, cervical, esophageal, endometrial, hepatobiliary, kidney, laryngeal, melanoma, oral, pancreatic, testis, or salivary gland cancer. A sample of the cancer diagnosis composition is shown in Figure A1.

Nearly half of the respondents were diagnosed between the ages of 50 and 69 (48.6%) and most respondents had their disease diagnosed between Stages II and IV (82.4%). The percentage distribution of respondents’ ages at diagnosis as well as the various diagnostic methods used to diagnose respondents’ conditions are detailed in Table A2 and are stratified by stage at diagnosis. The diagnostic methods that were reported by respondents but were not listed among the response options included colonoscopy, CT/MRI, mammogram or ultrasound, fecal immunochemical test (FIT), hysterectomy, emergency room visit, physical exam, and endoscopy. Most respondents’ (63.9%) cancers were diagnosed following more than one diagnostic method. Furthermore, 69% of the respondents reported having one or more biopsies to further investigate the makeup of their tumours.

While 29.2% had been tested for hereditary cancer syndromes, such as Lynch syndrome, or had conducted genetic panels assessing heritable cancer risk, of those who were tested, 4.2% tested positive for a hereditary cancer syndrome. The remaining respondents had not been tested for hereditary cancer syndromes (70.8%).

**Figure A2:**
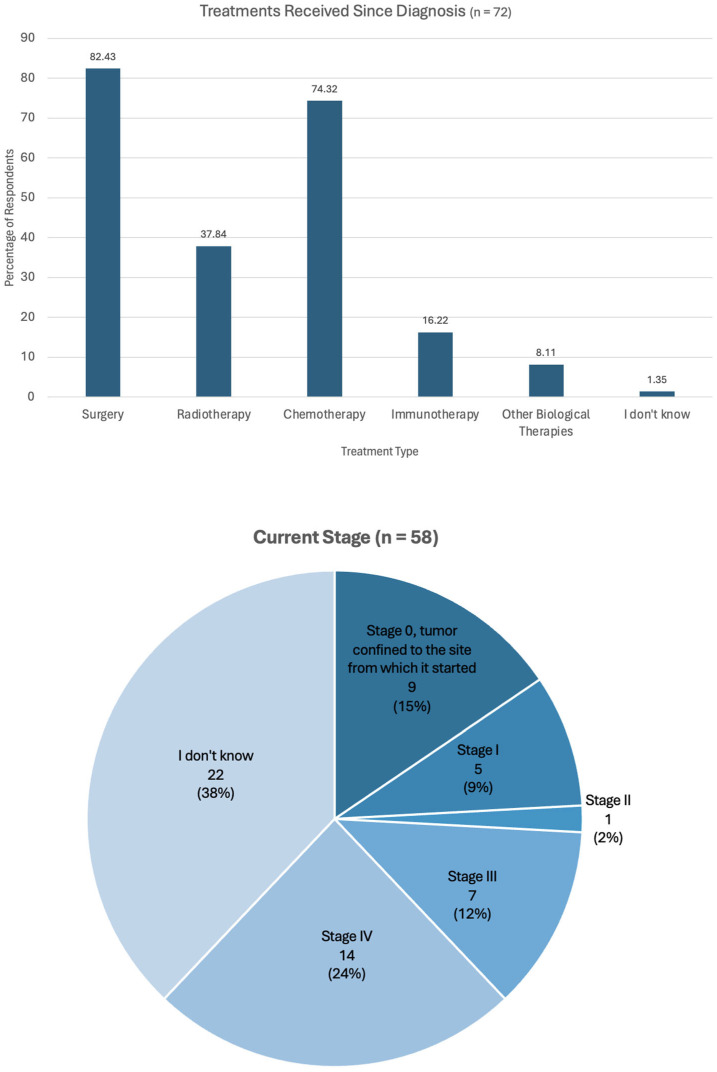
Cancer treatments received since diagnosis. Note, most respondents underwent more than one treatment following their diagnosis.

The two most reported treatments received since diagnosis were surgery to physically remove the cancer (82.4%) and chemotherapy (74.3%). Most respondents underwent multiple treatments since their diagnosis (90.5%), with only 9.4% of the respondents having undergone only one treatment (surgery). A total of 21.6% of the respondents had undergone surgery, radiotherapy, and chemotherapy, and 52.7% of the respondents had undergone a combination of two of the three. Other reported treatments that had been received but were not listed in the response options included hormone/androgen deprivation therapy, embolization, SSA medication, targeted molecular therapy, PARP inhibitor therapy, and autologous stem-cell transplant. The percentage distribution of treatments received since the diagnosis of the sample is shown in Figure A2.

#### 3.1.3. Biomarker Knowledge

**Figure 1:**
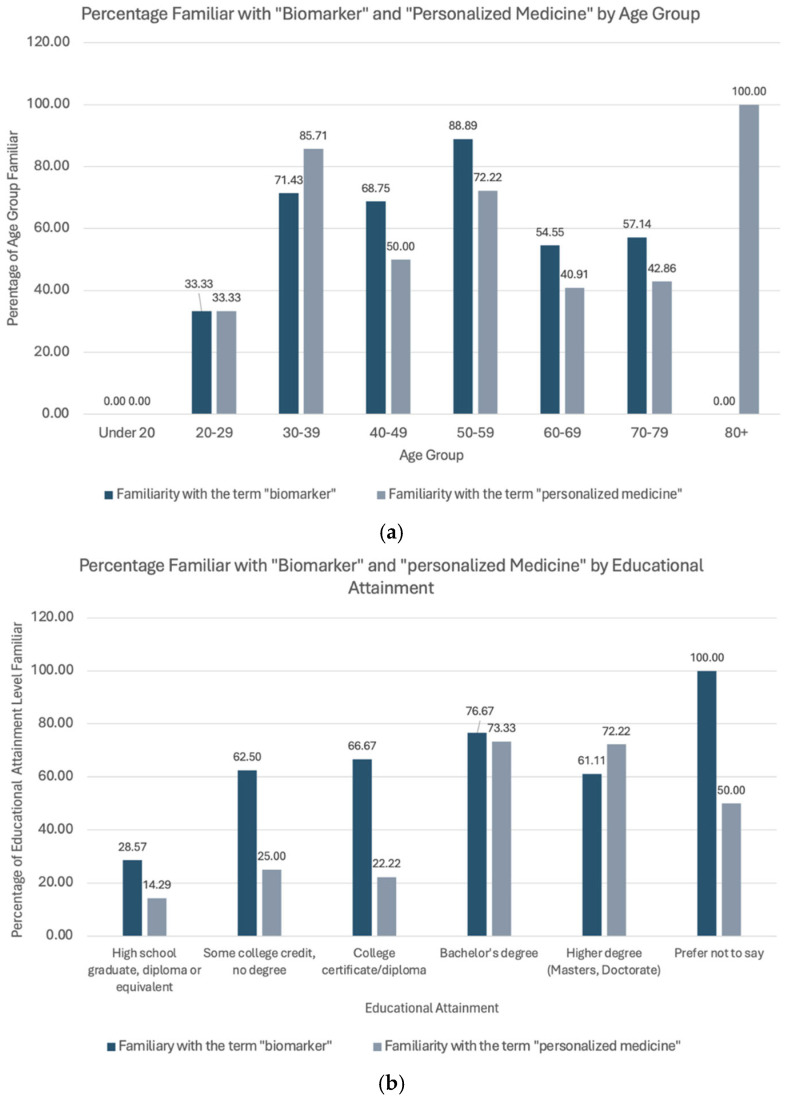
(**a**) The percentage composition of the respondents who reported being familiar with the terms “biomarker” and “personalized medicine”, stratified by age group. (**b**) The percentage composition of respondents who reported being familiar with the terms “biomarker” and “personalized medicine”, stratified by educational attainment.

Of 70 respondents, 70% were familiar with the term “biomarker” and 58.6% with the term “personalized medicine”. The percentages of the respondents who were familiar with the terms “biomarker” and “personalized” medicine, stratified by age group and educational attainment, are shown in Figure 1. The composition of the respondent pool who reported being familiar with the terms is shown in Table A3.

Of 70 respondents, 83% had heard of biomarker testing, while 17% had never heard of biomarker testing. Most respondents (20%) first heard the term “biomarker testing” through interactions with their oncologist and others first heard the term from other healthcare professionals (5.7%). While 30% of the respondents first heard it through the media, the internet, or through family and friends. The remaining respondents first heard of it through patient groups and from other cancer patients (11.4%). Furthermore, 41.4% of the respondents first learned about biomarkers from their care teams either at diagnosis or upon treatment selection. The composition of responses regarding where respondents first heard of biomarker testing, were stratified by the stage at which the respondents first learned of biomarkers from their care teams is shown in Table A4.

**Figure 2:**
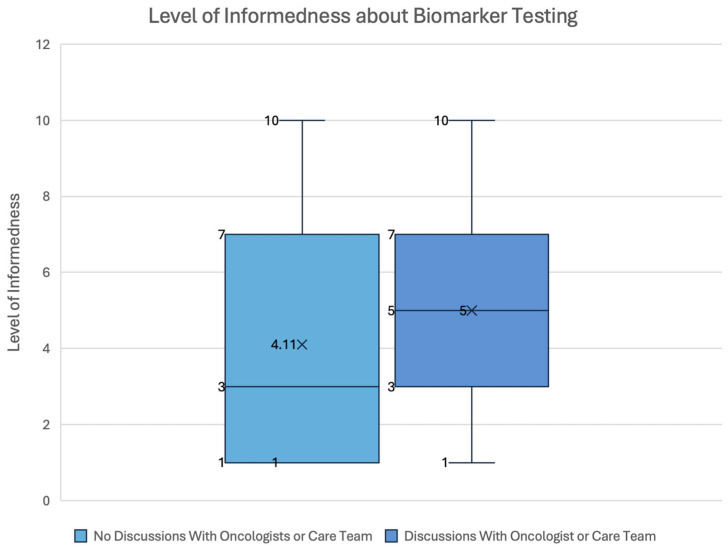
Level of informedness about biomarker testing and its impact on cancer treatment, between respondents who had spoken to their oncologists and respondents who had not.

Respondents were asked to rank their level of informedness about biomarker testing and its potential impact on their cancer treatment from 1 to 10, with 1 being “Not informed at all”, and 10 being “Very well-informed”. Among 71 respondents, the average level of informedness was 4.3 (±3 OR SD = 3) out of 10. The sample (71 respondents) was divided into two groups, one of which comprised respondents who had discussions with their oncologist or care team, either prior to the start of treatment or prior to any discussions about their treatment plan, and the other comprised patients who had no such discussions. The group that had discussions with their oncologists or care team demonstrated a higher level of informedness about biomarker testing, on average. The groups’ spread of informedness ranking is shown in Figure 2.

Among 70 respondents, the majority of respondents answered they were “unaware” when asked if they were aware that biomarkers can help determine the best cancer treatment for them, upon their diagnosis (62.9%). While 25.7% of the respondents reported being “somewhat aware” and 18.1% responded that they were “fully aware”. A breakdown of the sample’s awareness levels, stratified by age group and gender, is shown in Table A5.

Furthermore, Kendall’s tau coefficient was used to assess if there is an association between educational attainment and level of awareness that biomarkers could help determine the best therapeutic course for them upon diagnosis. Kendall’s tau coefficient analysis revealed a statistically non-significant, fairly weak correlation of 0.01135 [95% CI, *p* = 0.9051] between the two variables. Furthermore, the analysis also revealed a statistically non-significant, fairly weak, and negative correlation of −0.1241 [95% CI, *p* = 0.9051] between age at diagnosis and level of awareness that biomarkers could help determine the best therapeutic course for them upon diagnosis. These analyses indicate that there was no meaningful association between educational attainment, age at diagnosis, and the level of awareness that biomarkers could help determine the best therapeutic course, suggesting awareness of biomarkers’ role was not influenced by these factors.

#### 3.1.4. Biomarker Testing Informing Treatment

Of 55 respondents, 67.3% reported that neither their oncologist nor medical team explained biomarker testing prior to the start of their treatment. Furthermore, 63.6% of the respondents were not offered biomarker testing by their oncologist prior to discussions about their treatment plan, compared to 20.8% who were offered biomarker testing. The remainder of the sample responded that they were unable to recall, or that they had no treatment plan yet.

Of the 43 respondents whose physicians did not order biomarker testing, 65.1% did not request that their physician order a biomarker test while 27.9% did request that their physician order a biomarker test. The most common reason for not requesting biomarker testing themselves was “I did not know about it, to request it”, which 64.3% reported, while 21.4% reported that “I trust my physician knows best”. Kendall’s tau coefficient was used to assess if there was an association between the stage of cancer at diagnosis and the reason for not requesting biomarker testing. Kendall’s tau coefficient analysis revealed a statistically non-significant, fairly weak correlation of 0.1568 [95% CI, *p* = 0.354734] between the two variables, indicating that the stage of cancer at diagnosis did not have a meaningful association with the reason for not requesting biomarker testing.

**Figure 3:**
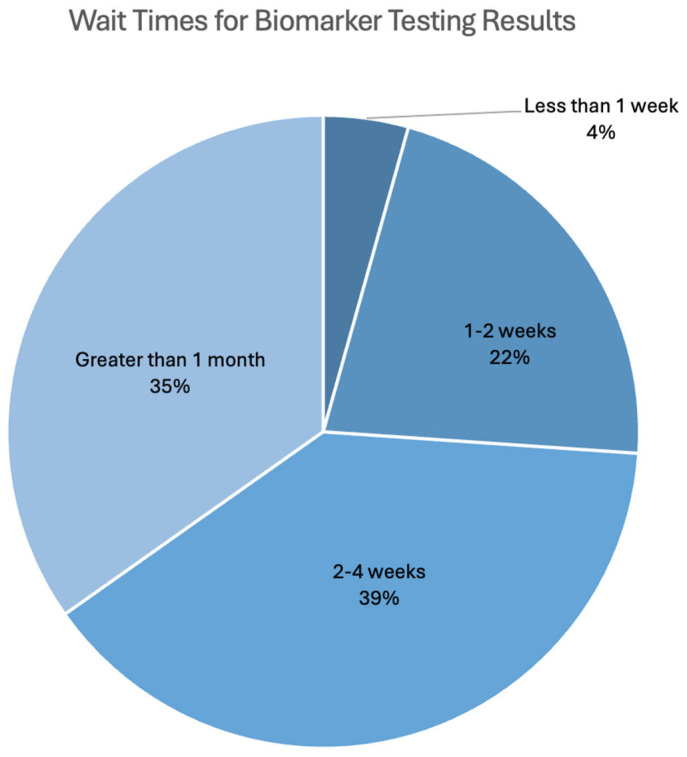
Waiting times for results after the doctor ordered biomarker testing.

**Figure A3:**
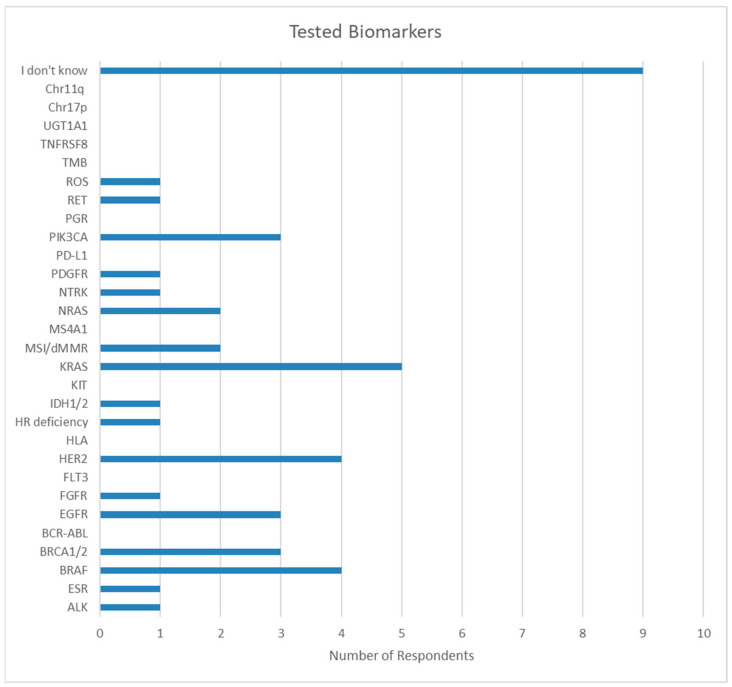
Biomarkers tested, as reported by patients. Other biomarkers that were tested for, but not listed included hormone receptor ER/PR, translocation biomarkers, homologous recombination repair deficiency (HRD), Inhibin b, antimullerian hormone (AMH), CA125, MutL protein homologue 1 MLH1, and carcinoembryonic antigen (CEA).

Furthermore, among 55 respondents, 54.5% reported having their biomarkers tested, while 38.2% did not, and 7.3% could not recall. The majority of the respondents could not recall which biomarkers they tested positive for (15%), while others reported that they tested positive for biomarkers such as KRAS, (8%), HER2 (6.5%), and BRAF (6.5%). The reported biomarkers tested for are shown in Figure A3. Among the 36 respondents who reported that their doctor ordered biomarker testing, 55.6% also reported that their doctors shared their results with them, 13.9% reported that their doctor did not share the results with them, and 5.6% reported receiving their results from their genetics department or accessing their results online. Waiting times for biomarker testing results are shown in Figure 3.

Regarding the stage at which biomarker testing was offered to explore therapeutic options, the largest portion of the sample was offered testing at Stage IV (27.3%). Spearman’s rho association coefficient was used to assess if there was an association between stage upon being offered biomarker testing and age group. Spearman’s rho association analysis revealed a statistically non-significant relationship and a fairly weak and a negative correlation of −0.08044 [95% CI, *p* = 0.4957] between the two variables, indicating that the stage at which biomarker testing was offered did not have a meaningful association with age group.

**Figure 4:**
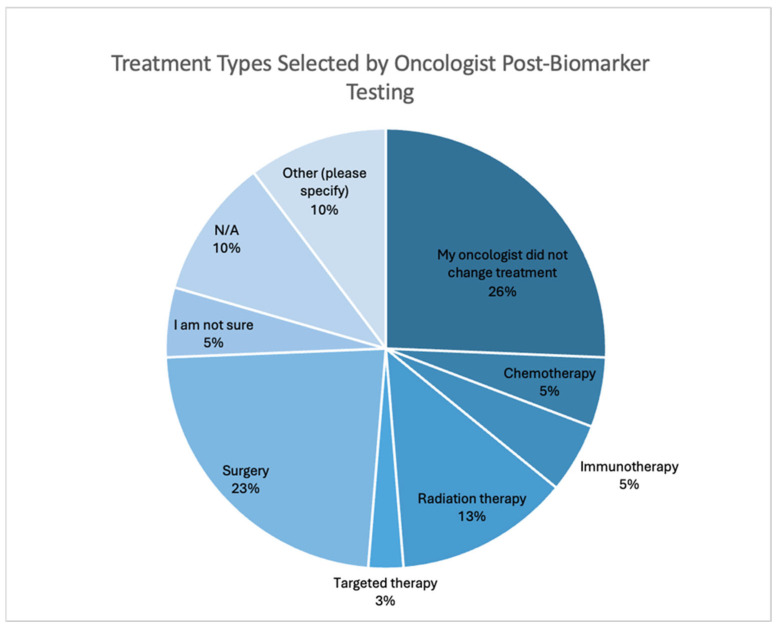
The types of treatment(s) selected by the respondents’ oncologists after they had biomarker testing. The types of treatment(s) that were selected but not listed among response options include hormone therapy, maintenance medication (post-chemotherapy), and panitumumab.

Furthermore, the greatest portion of the sample was undergoing/had undergone surgery when they had their biomarkers tested (50%). The percentage composition of the sample in terms of the stage when they were offered biomarker testing for therapeutic option exploration and the types of treatment respondents were undergoing is shown in Table A6. Out of 32 respondents, half reported that their oncologist changed their treatment after they had completed biomarker testing (50%). None of these respondents were in Stage 0 or III, while 12.5% were in Stage I and Stage IV, respectively. Figure 4 shows the types of treatments that were selected post testing.

Of the 16 respondents who had their treatment changed by their oncologist, 43.8% reported that their newly selected treatment was able to shrink/control their cancer and/or limit its metastases. Fischer’s exact test was used to assess if there is a relationship/association between pursuing biomarker testing and whether the newly selected treatment was able to shrink/control the patient’s cancer. The test revealed a statistically non-significant relationship [95% CI, *p* = 0.3137] between the two variables.

**Figure 5:**
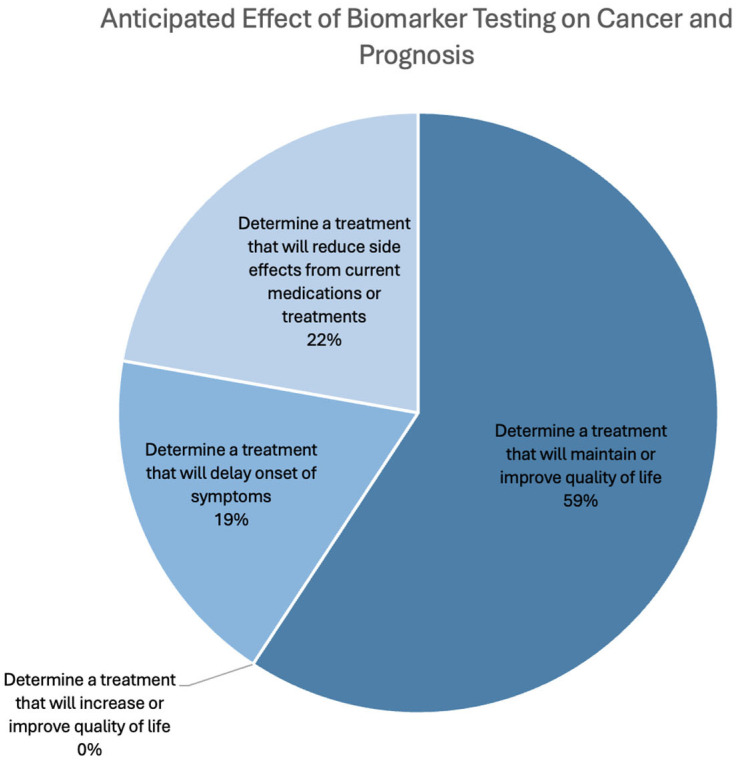
The effects respondents expected and/or hoped that biomarker testing results would have on their cancer and their prognoses.

Furthermore, of 35 respondents, only 5.7% were directed to a different cancer centre in order to receive their newly selected cancer treatment. Similarly, among 35 respondents, 5.7% were able to access clinical trials due to biomarker testing. The respondents were divided on whether biomarker testing helped them identify the right personalized treatments, as just over half (55%) answered “Yes”, while the remainder responded “No” (45%). Furthermore, 66.7% of the respondents felt that it was “Very important” to know their biomarkers (66.7%), and only 4.7% reported that it was “Not important”. Regarding the anticipated impact of biomarker testing on the respondents’ cancer conditions, most expected or hoped that it would maintain or improve their quality of life by determining a suitable treatment. The sample composition of the expected impact of biomarker testing is shown in Figure 5.

#### 3.1.5. Difficulties Experienced During Biomarker Testing

**Figure 6:**
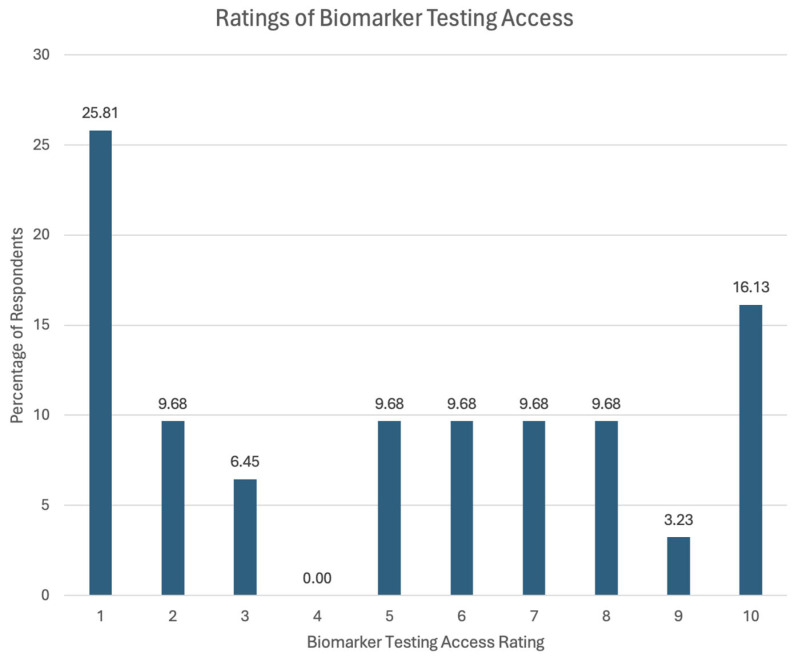
The distribution of sample ratings on access to biomarker testing at their institution. The mean rating was 5.06.

When asked to rate their overall experience with access to biomarker testing at their institution, 31 of the respondents provided their ratings on a scale of 1–10. A rating of 1 indicated “very limited/restrictive” access and a rating of 10 indicated “very appropriate/fair” access. The distribution of the sample’s ratings is shown in Figure 6. Among the 31 respondents, 25.8% indicated they experienced “very limited/restrictive” access while 16.1% of the respondents indicated they experienced “very appropriate/fair” access. The average rating from the respondents was 5.06.

**Figure 7:**
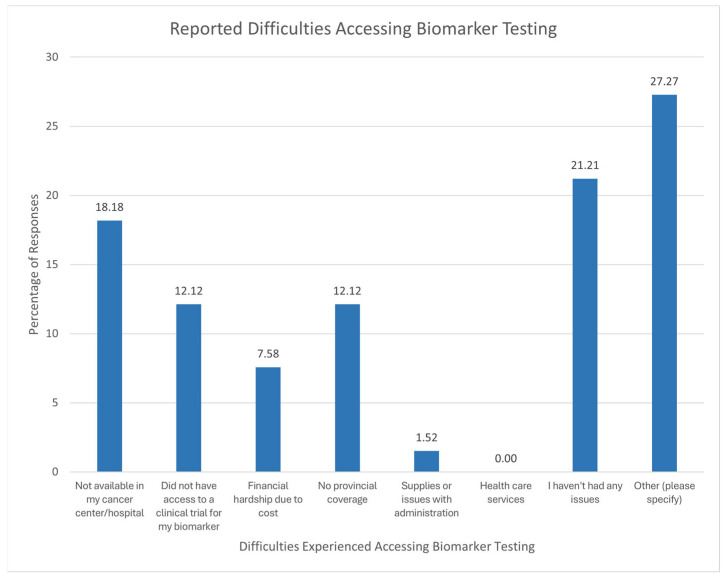
Reported difficulties regarding accessing biomarker testing. Other reported difficulties that were not listed among response options include insufficient biopsy samples and the unavailability of testing in the respondent’s area. Frequently reported difficulties were related to a lack of knowledge and awareness of biomarker testing.

Of 28 respondents, 27.3% reported incurring costs for biomarker testing. These respondents incurred costs ranging from $300 to $7500 over the course of their biomarker testing. Other difficulties regarding accessing biomarker testing reported by the respondents (66 respondents) are shown in Figure 7.

### 3.2. Thematic Analysis

From a self-management perspective, discussions brought up by the patients and caregivers were grouped into two themes. Regarding the first theme, *person-oriented attributes*, the responses revealed that active participation in care was intimately related to care responsibility, meaning that those who reported being responsible of their care actively took their care in their hands. For instance, except for one patient who was not aware of the clinical potential of biomarker testing, the rest of the participants sought information and were familiar with its associated benefits—even when in some cases they had not even been tested. For the participants, responsibility of care was as well associated with assuming the costs of such therapy, being accountable for the decisions taken regarding biomarker testing, and coping with adversity. The latter was associated with emotional management—and more specifically, seeking peace of mind after taking a decision based on the consideration of several treatment options.

Regarding the second theme, *person–environment-oriented attributes*, two ideas were brought up. First, informed patients were able to make informed decisions, which in turn played an important role in emotional coping. Information was valued to the point that some patients initiated activities such as blogs to inform peers. Also, information was deemed to be the most important component of the patient–physician partnership as it constituted being aware—on both sides, of facts and possibilities, that enhanced decision-making. Nevertheless, participants in this study revealed that they were not receiving the information they needed from their physicians. Thus, some participants reported having completed a search on their own about biomarker testing, while others expressed their frustration with not being aware of biomarker testing possibilities.

## 4. Discussion

This study investigated the knowledge, awareness, and experiences of patients and caregivers regarding biomarker testing for cancer care in Canada. Among the respondents, a considerable portion reported undergoing biomarker testing and having their treatment plans adjusted accordingly with positive results. Despite the numerous benefits associated with biomarker testing, cancer patients across Canada encounter many challenges that impede their access to these tests including a lack of knowledge and financial limitations. Thematic analysis of patient experiences underscores the role of biomarker testing in treatment decisions, emphasizes the importance of a patient-centred approach to care plans, and highlights the need for standardized biomarker testing guidelines at the institutional and national levels.

### 4.1. Main Findings

The survey results suggest there is evidence of moderate familiarity with the terms “biomarker testing” and “precision medicine”. Notably, the level of familiarity with the term “biomarker” is higher than with “precision medicine”. This trend suggests an increasing awareness of these terms since the previous iteration of the Get Personal Patient and Caregiver Survey, which was conducted in 2021, with a consistent pattern of greater familiarity with “biomarker” [27]. Among the age groups, those between 50 and 59 years demonstrated the highest familiarity, aligning with the targeted ages for colorectal cancer screening [28]. Higher levels of education were related to higher familiarity among the respondents. Despite moderate awareness of the terms biomarker and precision medicine among the respondents, a significant proportion of the respondents remain unfamiliar with the concepts of biomarker testing and its potential impacts on cancer treatment. Thus, these survey results highlight a persisting gap between awareness and deeper knowledge of the implications associated with biomarker testing and precision medicine.

Among the respondents familiar with biomarker testing, oncologists and other healthcare providers were the primary sources of information. These results coincide with other studies that identified oncologists and care teams as major information providers [29]. The survey results also show that engaging in conversations with healthcare professionals improved patients’ self-rankings of informedness, highlighting the importance of discussions with their care teams [29]. Many respondents first learned of biomarker testing at diagnosis or when selecting their treatment. However, a significant proportion of patients were not informed of biomarker testing at any point before or during their treatment. While the level of information about biomarker testing shared by oncologists is moderate according to the respondents, there are still large gaps in the proportions of patients who engage in discussions with their care teams. Therefore, these survey results reinforce the importance of educating patients to ensure they are aware of all their treatment options [30].

Moreover, survey responses align with previous research indicating that patients want to be involved in their decision-making [31]. The qualitative analysis allowed us to uncover that patients perceived a gap in patient-centred care. Feelings of regret and frustration, or experiencing dismissal from healthcare providers, were evident among the survey respondents. According to the respondents, information regarding potential genetic information, disease progression, treatment options, and future implications gives patients a sense of control and contributes to individual empowerment. As suggested in previous research, gaining any sense of control over difficult situations has positive impacts on a patient’s well-being and can contribute to better health outcomes, such as improved survival [32]. Furthermore, patients with a better understanding of biomarkers and precision medicine are likely to be more willing to take part in open discussions with their care teams [20].

When examining the impact of biomarker testing from the perspective of cancer patients and caregivers, survey results indicated that testing strongly influenced treatment decisions. Notably, a significant proportion of respondents reported that their new treatment led to either shrinking or controlling their cancer, further emphasizing the positive impacts of biomarker testing. These outcomes included access to a wider range of treatment options, changes to maintenance medication, and meeting eligibility criteria to enrol in clinical trials.

Overall, patients reported moderate experiences with biomarker testing but were impacted by multiple challenges throughout the testing process. The main challenges include a lack of standardized testing guidelines and infrastructure, long turnaround times, gaps in patient-centred care, and financial limitations. Similarly to the previous Patient and Caregiver Survey we conducted in 2021, a lack of testing availability and access to a relevant clinical trial emerged as major concerns [27]. Access to and the availability of biomarker testing are inconsistent across Canada and depend on multiple factors, creating disparities for patients. Incorporating standardized testing guidelines and practices for cancer care centres across Canada would provide patients with consistent testing access that is not dependent on factors outside their control [16,33].

Survey respondents also indicated a need to include biomarker testing as part of the standard practice of care. Expanding the array of cancers that can be investigated with biomarker testing holds promise for improving outcomes for more patients. However, limitations in healthcare provider knowledge and a lack of infrastructure to support an increase in biomarker testing would need to be overcome. Finally, advocacy groups are not available for all cancer types [21]. Without established community groups to inform patients of biomarker testing, those with less common cancers are at a disadvantage in terms of access and advocacy.

In addition to a lack of consistent guidelines across Canada, infrastructure issues include logistical challenges such as lengthy turnaround times for testing results. Among the survey respondents, most reported a wait time of at least four weeks for biomarker testing results. Long turnaround times for results can negatively impact patients in various ways. As highlighted in previous findings, increased delays in time to treatment can result in heightened feelings of anxiety and worse outcomes [34]. Improvements to infrastructure should be prioritized in healthcare systems across Canada to address inconsistencies in the biomarker testing process and to improve patient experiences [22].

Respondents expressed disappointment with the limited discussions with their healthcare provider, indicating a desire for a more patient-centred approach to care. Participants also noted that they were required to self-advocate for pursuing biomarker testing and faced difficulties in holding open discussions about the process. Since oncologists and other healthcare providers serve as the main resource for information, many patients are prevented from learning about biomarker testing if their healthcare providers do not engage in conversations about different treatment options [23]. Additionally, respondents expressed regret for not asking their healthcare provider about biomarker testing, reflecting an inclination to discuss treatment options further, but hesitancy often interfered. Engaging in patient-centred care should be a priority to ensure that patients and healthcare providers can build a strong relationship, as these approaches have been associated with increased trust and healthier patient outcomes [35].

Financial toxicity, including issues with coverage and access, is another barrier to biomarker testing reported in this survey. As noted in the literature, financial coverage is a major factor where inequality can arise [36,37]. Financial support for biomarker testing costs varies widely across provinces, impacting whether patients can proceed with the process [22]. Costs associated with cancer can vary depending on multiple factors such as cancer type, private insurance, employment status, and geographical location [38]. Direct costs include examples such as transportation fees to testing facilities, whereas indirect costs can arise from lost income from taking time off due to illness [37]. Additionally, provinces have different guidelines on which associated drugs are eligible for coverage which can cause further disparities for patients [39].

### 4.2. Strengths and Limitations

A major strength of this survey is that it provides a unique Canadian patient perspective on the gaps and opportunities associated with biomarker testing for cancer care, but there are several limitations. Firstly, limitations in the study’s demographics could impact the generalizability of the results. Survey respondents were predominantly characterized by above-average education levels, potentially influencing higher levels of patient education and awareness of biomarker testing. Moreover, there is an overrepresentation of colorectal cancer patients, which may skew perspectives, making them not fully representative of the broader cancer population. Other demographic biases include a higher proportion of female respondents and a large representation of residents from Ontario and Quebec. The small sample size in terms of the number of participants may also further influence the generalizability of these findings.

Inferential statistics were conducted during the analysis, but the correlations generated were found to be weak and non-significant (*p* > 0.05). Additionally, the sample size was small (74 responses), which can limit the statistical strength of the correlations presented and lead to inaccurate estimates [40].

In terms of survey methodology, recall and self-reporting biases can impact the internal validity of the results. This study included patients who had undergone biomarker testing and their caregivers, so respondents may have inaccurately recalled past experiences, potentially leading to recall bias. Additionally, this study included self-reported information from survey participants, which can lead to self-reporting bias and impact the validity of the descriptive and inferential statistics [41]. Furthermore, this survey did not include questions that addressed ethnicity or status as a visible minority, nor did it distinguish between unemployment and retirement status. Thus, disparities in biomarker testing access that are related to these factors are not completely examined within this survey.

Moreover, the survey did not include a section where respondents could define what they interpret and understand from the terms “biomarker” and “biomarker testing”, although the survey questions focused on familiarity with these terms. In future iterations of this survey, the aforementioned limitations will be addressed to ensure a comprehensive understanding of the study population and interpretation of these terms. Additionally, the open-ended questions provided participants with a limited opportunity to express their perspectives. To gain a better understanding of patient and caregiver perspectives, an in-depth study is needed, which could comprise a shorter version of an online qualitative survey and/or interviews and focus groups [24].

## 5. Conclusions

This study provides an overview of the perspectives and experiences of patients and caregivers regarding biomarker testing across Canada. The survey results indicate that patients are aware of biomarker testing and precision medicine but are challenged by a lack of further knowledge of the implications. While those who underwent biomarker testing reported positive outcomes with biomarker testing, the inequalities across Canada are still apparent. Patients continue to face challenges in terms of knowledge gaps, interprovincial variations in testing access, infrastructure limitations, and financial barriers. Qualitative analysis of patient experiences further highlighted a strong desire for patient-centred care and collaborative decision-making.

Future surveys should continue to assess biomarker testing knowledge and experiences among patients and caregivers, while also expanding the assessment of sociodemographic factors that may contribute to disparities in access. These survey findings will be vital for informing stakeholders of the importance of developing standardized testing guidelines that reduce inequalities among patients. Additionally, future studies should incorporate a qualitative component, such as interviews or focus groups, to gain deeper insights into patient and caregiver perspectives.

## Data Availability

The data presented in this study are available on request from the corresponding author. The data are not publicly available due to privacy restrictions.

## Supplementary Materials

The following supporting information can be downloaded at: https://www.mdpi.com/article/10.3390/curroncol32060292/s1. Get Personal: Patients and Caregivers Experiences and Knowledge with Biomarker Testing in Canada 2023 Survey.

## Appendix A

**Table A1:** The Demographics of the Survey Pool.

Demographic	Respondents (n)	Percentage (%)
*Gender*		
Female	60	81.08
Male	14	18.92
*Age (years)*		
<20	0	NA
20–29	3	4.05
30–39	7	9.46
40–49	16	21.62
50–59	18	24.32
60–69	22	29.73
70–79	7	9.46
80+	1	1.35
*Residence*		
Alberta (AB)	4	5.41
British Columbia (BC)	10	13.51
Manitoba (MB)	1	1.35
New Brunswick (NB)	2	2.70
Newfoundland and Labrador (NL)	2	2.70
Northwest Territories (NT)	0	NA
Nova Scotia (NS)	3	4.05
Nunavut (NU)	3	4.05
Ontario (ON)	30	40.54
Prince Edward Island (PEI)	0	NA
Quebec (QB)	20	27.03
Saskatchewan (SK)	2	2.70
Yukon (YT)	0	NA
*Employment Status*		
Full-time employment	23	31.08
Part-time employment	7	9.46
Not employed	44	59.46
*Educational Attainment*		
High school diploma or equivalent	7	9.46
Some college credit, no degree	8	10.81
College certificate or diploma	9	12.16
Bachelor’s degree	30	40.54
Higher degree (master’s or doctorate)	18	24.32
Preferred not to say	2	2.70

**Table A2:** Percentage distributions of respondents’ age at cancer diagnosis and diagnostic method, by stage at diagnosis.

Stage at Diagnosis	I	II	III	IV	I Don’t Know	Total
*Age at Diagnosis*						
Under 20 years	NA	NA	NA	1.39	NA	1.39
20–29 years	NA	1.39	1.39	1.39	NA	4.17
30–39 years	1.39	1.39	11.11	1.39	NA	15.28
40–49 years	1.39	6.94	5.56	6.94	1.39	22.22
50–59 years	5.56	6.94	8.33	2.78	1.39	25.00
60–69 years	4.17	4.17	8.33	6.94	NA	23.61
70–79 years	NA	NA	4.17	4.17	NA	8.33
80+ years	NA	NA	NA	NA	NA	NA
Total	12.50	20.83	38.89	25.00	2.78	100.00
*Diagnostic Method*						
Incidental finding/physical exam by family doctor	1.39	2.78	6.94	5.56	1.39	
Biopsy	5.56	13.89	25.00	11.11	1.39	
Reporting of symptoms and/or discomfort	2.78	12.50	16.67	18.06	NA	
Blood work	NA	4.17	15.28	5.56	NA	
Colonoscopy	1.39	4.17	9.72	1.39	1.39	
Other (please specify)	4.17	5.56	11.11	2.78	1.39	

**Table A3:** Composition of respondents who reported being familiar with the terms “biomarker” and “precision medicine”, stratified by age group as well as educational attainment.

Demographic		With “Biomarker”		With “Precision Medicine”	
	*Respondents*	*“Yes”*	*Percentage*	*“Yes”*	*Percentage*
*Age (years)*					
Under 20	0	0	NA	0	NA
20–29	3	1	33.33	1	33.33
30–39	7	5	71.42	6	85.71
40–49	16	11	68.75	8	50.00
50–59	18	16	88.88	13	72.22
60–69	22	12	54.54	9	40.91
70–79	7	4	5.147	3	42.86
80+	1	0	NA	1	100.00
*Educational Attainment*					
High school graduate, diploma or equivalent	7	2	28.57	1	14.29
Some college credit, no degree	8	5	62.5	2	25.00
College certificate/diploma	9	6	66.67	2	22.22
Bachelor’s degree	30	23	76.67	22	73.33
Higher degree (masters, doctorate)	18	11	61.11	13	72.22
Prefer not to say	2	2	100.00	1	50.00
Total	74	49		41	

**Table A4:** Composition of respondents by cancer stage when they learned of biomarkers from their care teams or by where they first heard of biomarker testing.

	Oncologist	Media/ Internet/ Family and Friends	Patient Groups/ Other Cancer Patients	Other Healthcare Physician	Other (Please Specify)	Total
At diagnosis	3	1	0	2	4	10
At treatment selection	4	8	1	0	1	14
When learning about clinical trials	0	0	1	0	1	2
When monitoring tumour recurrence	2	0	1	0	1	4
My cancer team has never discussed biomarkers	1	9	3	1	1	15
Other (please specify)	4	3	2	1	3	13
Total	14	21	8	4	11	58

**Table A5:** Level of awareness that biomarkers can help determine the best treatment, stratified by gender and age group.

	Unaware		Somewhat Aware		Fully Aware		
*Age (Years)*	No. of Respondents	Percentage	No. of Respondents	Percentage	No. of Respondents	Percentage	Total
<20	0	NA	0	NA	0	NA	0
20–29	0	NA	2	100.00	0	NA	2
30–39	3	42.86	2	28.57	2	28.57	7
40–49	11	73.33	3	20.00	1	6.67	15
50–59	9	52.94	4	23.53	4	23.53	17
60–69	14	66.67	7	33.33	7	NA	21
70–79	6	85.71	0	NA	0	14.29	7
80+	1	100.00	0	NA	0	NA	1
*Gender*							
Female	33	58.93	16	28.57	7	12.50	56
Male	11	78.57	2	14.29	1	7.14	14

**Table A6:** Percentage composition of sample regarding stage when patients were offered biomarker testing and if respondents had already received treatment upon offer.

	Number of Respondents	Percentage
*Stage when offered*		
0	0	NA
I	5	15.15%
II	5	15.15%
III	6	18.18%
IV	9	27.27%
I don’t know	3	9.09%
N/A	5	15.15%
*Type of treatment undergoing*		
Chemotherapy	9	34.61%
Immunotherapy	1	3.85%
Radiation Therapy	1	3.85%
Targeted Therapy	2	7.69%
Surgery	13	50.00%
