# Supplementary material for: Exploring Cancer Patients’ and Caregivers’ Perspectives and Knowledge Regarding Biomarker Testing in Canada
Source: Curr Oncol. 2025 May 22;32(6):292. doi: 10.3390/curroncol32060292 (PMC12191459; doi:10.3390/curroncol32060292)
Supplement: Supplementary file 1 [file curroncol-32-00292-s001.zip › curroncol-3580414-supplementary.pdf]

Supplemental Material

Get Personal: Patients and Caregivers Experiences and Knowledge with Biomarker Testing in Canada 2023

Purpose

Thank you for participating in this online survey for patients and caregivers. The purpose of this survey is to gather patients' experiences and knowledge related to biomarker testing in Canada. Insufficient patient knowledge surrounding diagnostic technologies including biomarker testing hinders cancer patients' access to new treatments and better clinical outcomes. This survey will help assess needs and gaps in Canadian personalized medicine by gathering patients' experiences with biomarker testing in cancer treatment centres across Canada. The ultimate goal is to improve access and quality to biomarker testing, in order to improve outcomes for cancer patients. Similar national surveys have been conducted in other countries, and this survey will allow the first Canadian specific data.

Colorectal Cancer Canada's Get Personal Program aims to educate patients and inform health policy about biomarker testing for use with companion diagnostics to increase access to personalized healthcare and precision medicines based on a patient's specific genomic profile.

The goal of the survey is to:

- Identify patients' knowledge and awareness of biomarker testing
- Describe patients' experiences with biomarker testing
- Describe difficulties and barriers patients face throughout the process biomarker testing

There are three sections in this survey:

Section 1: Personal Demographics and Cancer Diagnosis

Section 2 – Knowledge on Biomarker Testing

Section 3 – Experience with Biomarker Testing

Section 4 – Additional Information (Optional)

Instructions

Participation in this study is entirely voluntary. You can choose not to participate or discontinue participation at any time. The survey includes three main sections: personal demographics and cancer diagnosis; your knowledge of biomarker testing; and your experience with biomarker testing.

This survey can be completed by the patient and/or by the caregiver on behalf of the patient. This survey should take approximately 15 to 20 minutes to complete. Once you complete the questions and submit the survey, your response will be sent directly to the Colorectal Cancer Canada's Get Personal Program Manager.

All of your responses to this survey will remain anonymous and cannot be linked to you in any way. Once you submit your completed survey, there will be no way to withdraw your responses from the study because there is no mechanism to identify you.

Study data are returned to the Program Manager in a digital format that does not identify individual responses. The digital, non-identifiable data will be kept by the Program Manager on a password-protected computer. Combined or analyzed, non-identifiable data will be shared with the medical community and the public to increase awareness about this important public health issue.

There are no risks associated with this study. While you will not experience any direct benefit from participation, information collected in this study may benefit others in the future by contributing to our knowledge and understanding of the performance of biomarker testing across provinces and territories in Canada.

If you have any questions regarding the survey or Colorectal Cancer Canada's, Get Personal Program, please contact the Get Personal Program Manager, Patil Mksyartinian, at patilm@colorectalcancercanada.com.

To show our appreciation for the time spent on this survey, to opt in, please leave your mailing address in the appropriate field.

You will not be financially compensated for your participation however, to show our appreciation for the time spent completing the survey, 10 of the total respondents will randomly be selected to receive a $25 Amazon E-Gift Card. To ensure your survey responses remain anonymous, you will be asked to input your e-mail address for the purposes of entering to win an e-gift card. This identifying information will be stored separately, then destroyed once remuneration has been provided.

If you agree to participate, you are asked to fill out the survey provided below. By completing and submitting this survey, you are indicating your consent to participate in this study. There is no need for a signed consent to participate. Please complete the survey by September 30th, 2023. Your voice is so important.

1. "I agree that this information is being provided voluntarily and by providing this information I consent to its use by CCC for statistical purposes."
    - Agree
    - Disagree

Section 1: Personal Demographics and Cancer Diagnoses

2. What is your connection to cancer?
    - Patient undergoing treatment
    - Patient previously treated
    - No evidence of disease (no physical evidence of cancer on examination or imaging tests after treatment)
    - Caregiver on behalf of patient undergoing treatment
    - Caregiver on behalf of patient previously treated

3. In which part of Canada do you live?
    - Alberta
    - British Columbia
    - Manitoba
    - New Brunswick
    - Newfoundland & Labrador

- Northwest Territories
- Nova Scotia
- Nunavut
- Ontario
- Prince Edward Island
- Quebec
- Saskatchewan
- Yukon

4. How far do you live from the nearest cancer centre?
   - < 25 km
   - 25-50 km
   - 50-75 km
   - 75-100 km
   - 100-150 km
   - > 150 km
   - I am not sure

5. Which age group do you belong to?
   - Under 20 years
   - 20-29 years
   - 30-39 years
   - 40-49 years
   - 50-59 years
   - 60-69 years
   - 70-79 years
   - 80+ years

6. Which gender do you most identify with:
   - Male
   - Female
   - Other
   - Prefer not to say

7. What is the highest degree or level of education you have completed?
   - No schooling completed
   - High school graduate, diploma or equivalent
   - Some college credit, no degree
   - College certificate/diploma
   - Bachelor’s degree
   - Higher degree (Masters, Doctorate)
   - Prefer not to say

8. Are you currently working?
   - Yes, full-time
   - Yes, part-time
   - No

9. What is the cancer treatment centre or hospital where you received your care?
    - o Alberta Health Services
    - o BCCA—Vancouver Cancer Centre
    - o BCCA – Centre for the Southern Interior
    - o BCCA – Vancouver Island Cancer Centre
    - o BCCA – Fraser Valley Centre
    - o Brampton Civic Hospital William Osler
    - o CancerCare Manitoba
    - o Centre de Recherche sur le Cancer (Université Laval)
    - o CHUM
    - o CHU de Quebec
    - o CHUS
    - o Cross Cancer Institute
    - o Georges-Dumont
    - o Hôpital Maisonneuve-Rosemont
    - o Jewish General Hospital
    - o Juravinski Cancer Centre
    - o Kingston General Hospital
    - o Kleysen Institute for Advanced Medicine - Health Sciences Centre
    - o London Health Sciences Centre
    - o Markham Stoufville Hospital
    - o Moncton Hospital
    - o Mount Sinai Hospital
    - o MUHC
    - o Odette Cancer Centre
    - o Ottawa Hospital
    - o Princess Margaret
    - o QEII Health Sciences Centre
    - o Saskatoon Cancer Centre
    - o SickKids
    - o St. Joseph's Health Centre
    - o St. Michael's Hospital
    - o Sunnybrook Health Sciences Centre
    - o Toronto General Hospital
    - o Tom Baker Cancer Centre
    - o Trillium Health Partners
    - o Trois-Rivières
    - o University Health Network
    - o Vancouver General Hospital
    - o William Osler Health System
    - o Other (please specify) __________

10. What type of cancer were you diagnosed with?
    - o Bladder
    - o Brain/CNS
    - o Breast
    - o Cervix
    - o Colorectal

- Esophagus
- Endometrial
- Blood (Hematologic)
- Liver (Hepatobiliary)
- Kidney
- Larynx
- Lung
- Melanoma
- Oral
- Ovary
- Pancreas
- Prostate
- Thyroid
- Testis
- Salivary gland
- Other (please specify) ________

11. Have you been tested for hereditary cancer syndromes*?

*The most common inherited syndrome that increases a person's risk for colon cancer is Lynch syndrome (Hereditary Non-Polyposis Colorectal Cancer) which also leads to high risk of endometrial cancer, as well as cancers of the ovary, stomach, small intestine and others.
Other hereditary cancer syndromes include Li-Fraumeni syndrome.

- No
- Yes (please specify) ________

12. Have you ever tested positive for any of the hereditary cancer syndromes*?

*The most common inherited syndrome that increases a person's risk for colon cancer is Lynch syndrome (Hereditary Non-Polyposis Colorectal Cancer) which also leads to high risk of endometrial cancer, as well as cancers of the ovary, stomach, small intestine and others. Other hereditary cancer syndromes include Li-Fraumeni syndrome.

- No
- Yes (please specify) __________

13. What is the date of diagnosis? (day/month/year) example: 27/01/2019

____

14. What was the stage of your disease at diagnosis?
    - Stage 0, tumor confined to the site from which it started
    - Stage I
    - Stage II
    - Stage III
    - Stage IV
    - I don't know

15. If applicable, what is the current stage?
    - Stage 0, tumor confined to the site from which it started
    - Stage I
    - Stage II

- Stage III
- Stage IV
- I don’t know

16. How old were you when you were first diagnosed with cancer?
    - Under 20 years
    - 20-29 years
    - 30-39 years
    - 40-49 years
    - 50-59 years
    - 60-69 years
    - 70-79 years
    - 80+ years

17. What method(s) were used to diagnose the cancer? Check all that apply
    - Incidental Finding / Physical Exam at Family Doctor
    - Biopsy
    - Reporting of symptoms and/or discomfort
    - Blood work
    - Other (please specify) __________

18. Did you have one or more biopsies to further investigate the make-up of your tumour
    - Yes
    - No

19. What treatment(s) did you receive since diagnosis? Check all that apply
    - Surgery (cancer physically removed)
    - Radiotherapy (high doses of radiation to "kill" cancer cells or shrink tumours)
    - Chemotherapy (therapy to "kill" cancer cells or shrink tumours)
    - Immunotherapy (biological therapy that helps the immune system to fight the cancer)
    - Other biological therapies
    - I don't know
    - Other (please specify) ____________

Section 2: Knowledge on Biomarker Testing
Biomarkers (short for biological markers) are molecules found in body tissues and fluid, including tumour tissue and blood. Biomarker testing involves the collection of a sample of your tumour, either tissue biopsy or liquid biopsy (blood sample test), that will later be sent to a certified pathologist. Biomarker testing allows health care providers to uncover specific genetic and molecular characteristics which can help them refine and provide a personalized treatment plan for you. Biomarker testing is also known as molecular testing and tumour profiling.

20. Prior to reading the excerpt above, were you:
    (a) Familiar with the term “biomarker”?
    - Yes
    - No

21. Prior to reading the excerpt above, were you:
    (a) Familiar with the term “personalized medicine”?

- Yes
- No

22. Where did you first hear about biomarker testing?
    - Oncologist
    - Family/friends
    - Media
    - Internet
    - Other cancer patient
    - Patient group
    - Other healthcare physician
    - I have never heard of biomarker testing
    - Other (please specify) ___________

23. On a scale of 1-10, with 1 being "not informed at all" and 10 being "very well-informed", how well were you informed about biomarker testing and how it can impact your cancer treatment?
    - *Scale from 1-10

24. When you were diagnosed, were you aware that biomarkers can help determine the best treatment for you
    - Fully aware
    - Somewhat aware
    - Unaware

25. Please indicate at what stage in your cancer care did you first learn about biomarkers from your care team:
    - At diagnosis
    - At treatment selection
    - When learning about clinical trials
    - When monitoring tumour recurrence
    - My cancer team has never discussed biomarkers
    - Other (please specify) _________

Section 3: Experience with Biomarker Testing

26. Did your oncologist or any other member of your medical team explain biomarker testing before your treatment started?
    - Yes
    - No
    - N/A

27. Did your oncologist offer you biomarker testing prior to discussions about your treatment plan?
    - Yes
    - No
    - I don't remember
    - N/A; no treatment plan yet

28. If no, did you request that your physician order a biomarker test for you?
    - Yes
    - No

- I don’t remember

29. If you didn't request a biomarker test from your physician, why did you not? Check all that apply
    - Fear of asking my physician
    - Did not know about it to request it
    - I trust my physician knows best
    - Other (please specify) _____________

30. Did you get your biomarkers tested?
    - Yes
    - No (skip to question 46)
    - I don’t remember

31. At what stage of cancer were you offered biomarker testing to explore your therapeutic options?
    - Stage 0, tumour confined to the site from which it started
    - Stage I
    - Stage II
    - Stage III
    - Stage IV
    - I do not know
    - N/A
    - Other (please specify) _____________

32. If your doctor ordered biomarker testing for you, did they share the results with you?
    - Yes
    - No
    - Other (please specify) _____________

33. Do you know which biomarker(s) you tested positive for? Check all that apply
    - ALK
    - ESR
    - BRAF
    - BRCA1/2
    - BCR-ABL
    - EGFR
    - FGFR
    - FLT3
    - HER2
    - HLA
    - HR deficiency
    - IDH1/2
    - KIT
    - KRAS
    - MSI/dMMR
    - MS4A1
    - NRAS
    - NTRK
    - PDGFR

- PD-L1
- PIK3CA
- PGR
- RET
- ROS
- TMB
- TNFRSF8
- UGT1A1
- Chr 17p
- Chr 11q
- I don't know
- Other (please specify) _______________

34. If your doctor ordered biomarker testing for you, around how long did it take for you to receive the results after the sample was taken?
    - Less than 1 week
    - 1-2 weeks
    - 2-4 weeks
    - Greater than 1 month
    - I don't remember

35. Were you already receiving cancer treatment when you got your biomarker tested? If so, what type(s)? Check all the apply
    - Chemotherapy
    - Immunotherapy
    - Radiation therapy
    - Targeted therapy
    - Surgery
    - Other (please specify) _______________

36. After you had your biomarkers tested, what treatment(s) did your oncologist select? Check all that apply
    - My oncologist did not change treatment
    - Chemotherapy
    - Immunotherapy
    - Radiation therapy
    - Targeted therapy
    - Surgery
    - I am not sure
    - N/A
    - Other (please specify) _________________

37. At what stage of cancer were you when your oncologist changed your treatment after biomarker testing?
    - Stage 0, tumour confined to the site from which it started
    - Stage I
    - Stage II
    - Stage III

- Stage IV
- Not applicable: I did not change treatments
- I am not sure
- Other (please specify) ____________________

38. Was this treatment able to shrink/control your cancer and/or spread of the disease to other organs (metastases)?
    - Yes
    - No
    - Partially
    - I don't know

39. Were you directed to a different cancer center in order to receive your cancer treatment?
    - Yes
    - No
    - N/A

40. Did biomarker testing help you access a clinical trial?
    - Yes
    - No (please explain) _____________________

41. Do you feel that biomarker testing helped you find the right personalized treatment?
    - Yes
    - No
    - If yes, specify what treatment __________________

42. What effect do you expect (or hope) that your biomarker test results will have on the cancer and your prognosis? Check all that apply
    - Determine a treatment what will maintain or improve quality of life
    - Determine a treatment that will increase overall survival
    - Determine a treatment that will delay onset of symptoms
    - Determine a treatment that will reduce the side effects from current medications or treatments
    - Other (please specify) ____________________

43. On a scale of 1-10, with 1 being "very limited/restrictive" and 10 being "very appropriate/fair", how would you rate your overall experience with access to biomarker testing at your institution?
    - *Scale from 1-10

44. Did access to biomarker testing allow you to fulfill or accomplish anything that you would have otherwise been unable to, had you not accessed it? (medically, socially, psychologically)? If yes, please explain.
    ____

45. Did you incur any costs for biomarker testing? If yes, please specify the amount:
    - No
    - I am not sure
    - Yes (please specify) ____________________

46. Have you experienced any difficulties accessing biomarker testing? Check all that apply
    - Not available in my cancer center/hospital
    - Did not have access to a clinical trial for my biomarker
    - Financial hardship due to cost
    - No provincial coverage
    - Supplies or issues with administration
    - Health care services
    - I haven't had any issues
    - Other (please specify) ___________________________

47. What would you change about your experience with biomarker testing? What could have improved the biomarker testing process?

    ____

Section 4: Additional Information

This section is optional and seeks to gather additional information. Please note that your name and any personal details that you may identify you will not be shared.

48. How important was it for you to know your biomarkers?
    - Very important
    - Somewhat important
    - Not important
    - Other (please specify) ____________________

49. Is there anything else about your experience with biomarker testing that you would like us to know and include? Please describe below.

    ____

50. Check all that apply:
    - I would like to enter the prize draw (for a chance to win 1 of 10 Amazon e-gift cards)
    - I would like to be contacted for qualitative interviews on my experience with biomarker testing
    - I would like to be contacted to provide feedback for this survey for quality improvement purposes
    - I wish to receive Colorectal Cancer Canada's patient support groups
    - I am interested in Colorectal Cancer Canada offering a support group to discuss my experience(s) with biomarker testing
    - I would like to share my story with cancer on Colorectal Cancer Canada's website (https://www.colorectalcancercanada.com/stories/)

51. If you selected any of the options above, please leave your email address here:
    - Please note, you will only be contacted regarding the option(s) that you selected.

    ____
